# Low-dose CT screening for lung cancer in Brazil: a study protocol

**DOI:** 10.1590/S1806-37132014000200016

**Published:** 2014

**Authors:** Ricardo Sales dos Santos, Juliana Franceschini, Fernando Uliana Kay, Rodrigo Caruso Chate, Altair da Silva Costa, Fernando Nunes Galvão de Oliveira, André Luiz Cavalcante Trajano, José Rodrigues Pereira, Jose Ernesto Succi, Roberto Saad

**Affiliations:** Center for Minimally Invasive Thoracic Surgery, Robotics & Bronchoscopy, Hospital Israelita Albert Einstein; and Principal Investigator, ProPulmão, São Paulo, Brazil; ProPulmão, São Paulo, Brazil; University of São Paulo School of Medicine Hospital das Clínicas; and Physician, Department of Diagnostic Support, Hospital Israelita Albert Einstein, São Paulo, Brazil; Department of Diagnostic Support, Hospital Israelita Albert Einstein, São Paulo, Brazil; Pediatric Thoracic Surgery Outpatient Clinic, Department of Thoracic Surgery, Federal University of São Paulo Paulista School of Medicine, São Paulo, Brazil; and Professor, Department of Thoracic Surgery, ABC School of Medicine, Santo André, Brazil; CLION/GRUPO CAM, Salvador, Brazil; Cardiopulmonary Institute, Salvador, Brazil; Portuguese Beneficent Hospital of São Paulo, São Paulo, Brazil; Department of Thoracic Surgery, Federal University of São Paulo Paulista School of Medicine, São Paulo, Brazil; Santa Casa School of Medical Sciences in São Paulo, São Paulo, Brazil

## To the Editor:

Because of the lack of studies aimed at screening for lung cancer (LC) in the Brazilian population, a project that is integrated into the Program for the Support of the Institutional Development of the Brazilian National Ministry of Health Unified Health Care System and whose objective is to evaluate the efficacy of low-dose CT (LDCT) scans of the chest in screening for LC was launched. The objective of the present letter was to describe the design and methods of the *Projeto de Detecção Precoce do Câncer de Pulmão* (ProPulmão, Project for Early Detection of Lung Cancer), which was approved by the Research Ethics Committee of the *Instituto Israelita de Ensino e Pesquisa do Hospital Albert Einstein* (Protocol no. CAAE 02087012.1.0000.0071).

For the development of the project, the final sample will comprise 1,000 individuals recruited as of 2013 via public calls in vehicles of communication in the greater metropolitan area of São Paulo, as well as via partnerships with other community care services. The sample size was calculated on the basis of previous international studies addressing this issue.^(^ ^1^ ^)^

The inclusion criteria are as follows^(^ ^2^ ^)^: having no respiratory symptoms; being in the 55-74 year age bracket; being a smoker with a smoking history of at least 30 pack-years or having been a former smoker for 15 years at most; and agreeing to participate in the study by giving written informed consent. The exclusion criteria are as follows: being unable to undergo CT scans; being pregnant; having previously undergone radiation therapy to the chest; and having severe chronic disease, such as cardiovascular disease, lung disease, liver disease, kidney disease, and metabolic disease.

The primary outcome measure is early diagnosis of LC. Nevertheless, participants will undergo a multidisciplinary evaluation for smoking-related diseases and infectious diseases that are common in Brazil, such as tuberculosis.

At the initial visit, demographic and smoking history data will be collected; health-related quality of life will be assessed by the Medical Outcomes Study 36-item Short-form Health Survey^(^ ^3^ ^)^; the presence of anxiety or depression will be determined by the hospital anxiety and depression scale^(^ ^4^ ^)^; and the presence of nicotine dependence in current smokers will be determined by the Fagerström test.^(^ ^5^ ^)^

**Chart 1:**
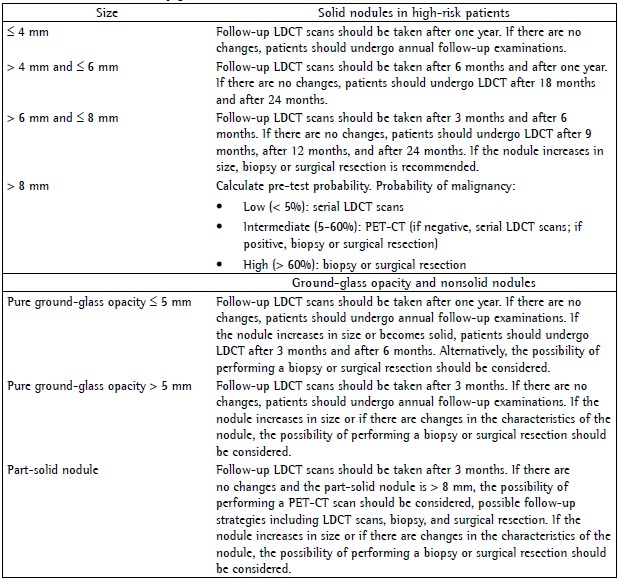
Follow-up strategies to monitor high-risk patients for solid nodules, ground-glass opacity, and nonsolid nodules, based on the National Comprehensive Cancer Network Guidelines for Lung Cancer Screening and on the Fleischner Society guidelines

After the initial evaluation, individuals will be referred for LDCT screening, the scans being analyzed by two radiologists with experience in thoracic diseases. Indeterminate pulmonary nodules = 4 mm in size will be evaluated by a medical team comprising radiologists, pulmonologists, and thoracic surgeons, who will decide on the follow-up strategy (Chart 1).

LDCT: low-dose CT; and PET-CT: positron emission tomography-CT. ^a^Adapted from the National Comprehensive Cancer Network,^(^ ^7^ ^)^ MacMahon et al.,^(^ ^8^ ^)^ and Patel et al.^(^ ^9^ ^)^

In cases of solid nodules > 8 mm in size, radiological features alone are not enough to distinguish between benign and malignant nodules. Therefore, it is important to estimate the clinical probability of malignancy. This estimation is known as pre-test probability and aids in reducing interobserver variability regarding the probability of malignancy. A multivariate logistic regression model developed at Mayo Clinic^(^ ^6^ ^)^ on the basis of six independent predictors of malignancy-including patient age (in years), being a smoker or former smoker, having a history of extrathoracic cancer diagnosed more than 5 years prior, nodule diameter (in mm), presence of spicules, and upper lobe involvement-will be used in the study.

After undergoing LDCT, all patients will return for a follow-up evaluation, in which the LDCT findings will be recorded and the follow-up strategy will be proposed. At that visit, current smokers will be referred to a smoking cessation program. Although participation in the program is encouraged, enrollment is voluntary.

**Figure 1:**
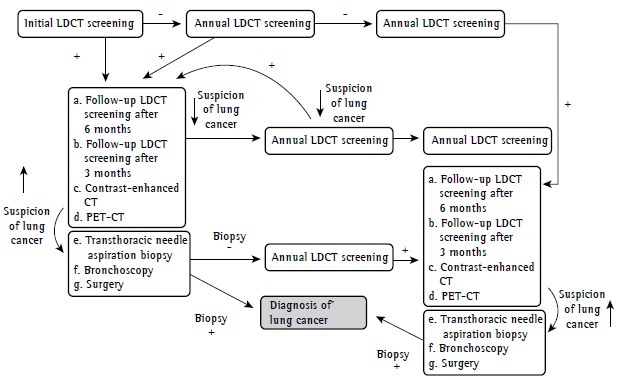
Flowchart of possible follow-up strategies. LDCT: low-dose CT; and PET-CT: positron emission tomography-CT.

Abnormal CT findings will be recorded on a specific form, analyzed by the expert panel, and classified on the basis of the level of suspicion of malignancy, follow-up strategies being subsequently decided on (Figure 1).

In cases of lung cancer, the nodules seen on the follow-up LDCT scans will be compared with those seen on the initial LDCT scans; the parameters for all CT scans will be the same, therefore allowing the examination of possible changes.

Subsequent visits, occurring in the second year of follow-up, will be conducted in accordance with the flowchart shown in Figure 1, specific findings in each individual in the previous year being taken into consideration (Chart 1).

The attending physician at the outpatient clinic will give the participants the results of the LDCT examinations. In addition, the medical team will inform the participants of the suspicion or diagnosis of LC.

After diagnostic confirmation and surgical treatment (when appropriate), patients will be referred for oncological follow-up via the Brazilian Unified Health Care System or the private health care system and will receive adjuvant therapy as medically indicated.

To date, there have been no studies of LDCT screening for LC in developing countries, in which the incidence of infectious diseases of the chest is higher. This raises many questions regarding the sensitivity and specificity of the method for LC screening.

The use of LDCT screening in Brazil is of fundamental importance because it will provide specific information for the validation of the method as a population screening tool for LC.
