# Supplementary material for: Low-dose CT screening for lung cancer in Brazil: a study protocol
Source: J Bras Pneumol. 2014 Mar-Apr;40(2):196–9. doi: 10.1590/S1806-37132014000200016 (PMC4083651; doi:10.1590/S1806-37132014000200016)
Supplement: Translation

# Translation: Rastreamento de câncer de pulmão por meio de TC de baixa dosagem no Brasil: protocolo de pesquisa

**Authors:** Ricardo Sales dos Santos, Juliana Franceschini, Fernando Uliana Kay, Rodrigo Caruso Chate, Altair da Silva Costa, Fernando Nunes Galvão de Oliveira, André Luiz Cavalcante Trajano, José Rodrigues Pereira, Jose Ernesto Succi, Roberto Saad

**Affiliations:** Centro de Cirurgia Torácica Minimamente Invasiva, Robótica e Broncoscopia, Hospital Israelita Albert Einstein; e Investigador Principal, ProPulmão, São Paulo (SP) Brasil; ProPulmão, São Paulo (SP) Brasil; Hospital das Clínicas, Faculdade de Medicina, Universidade de São Paulo; e Médico, Departamento de Apoio Diagnóstico, Hospital Israelita Albert Einstein, São Paulo (SP) Brasil; Departamento de Apoio Diagnóstico, Hospital Israelita Albert Einstein, São Paulo (SP) Brasil; Ambulatório de Cirurgia Torácica Infantil, Disciplina de Cirurgia Torácica, Escola Paulista de Medicina, Universidade Federal de São Paulo, São Paulo (SP) Brasil; e Professor, Disciplina de Cirurgia Torácica, Faculdade de Medicina do ABC, Santo André (SP) Brasil; CLION/GRUPO CAM, Salvador (BA) Brasil; Instituto Cardiopulmonar, Salvador (BA) Brasil; Hospital Beneficência Portuguesa de São Paulo, São Paulo (SP) Brasil; Disciplina de Cirurgia Torácica, Escola Paulista de Medicina, Universidade Federal de São Paulo, São Paulo (SP) Brasil; Faculdade de Ciências Médicas, Santa Casa de São Paulo, São Paulo (SP) Brasil

## Ao Editor,

Devido à ausência de estudos para o rastreamento do câncer de pulmão (CP) na população brasileira, foi iniciado um projeto com o intuito de avaliar a capacidade de rastreamento populacional do CP por meio de TC de baixa dosagem (TCBD) de tórax, integrado ao Programa de Apoio ao Desenvolvimento Institucional do Sistema Único de Saúde do Ministério da Saúde. O objetivo da presente carta foi descrever o delineamento e os métodos do Projeto de Detecção Precoce do Câncer de Pulmão (ProPulmão), o qual foi aprovado pelo Comitê de Ética em Pesquisa do Instituto Israelita de Ensino e Pesquisa do Hospital Albert Einstein (CAAE 02087012.1.0000.0071).

Para o desenvolvimento do projeto, a amostra final será composta por 1.000 indivíduos, os quais estão sendo recrutados desde 2013, a partir de chamadas públicas em veículos de comunicação da região metropolitana de São Paulo e de parcerias com outros serviços de atendimento à comunidade. O tamanho da amostra foi calculado com base em estudos internacionais prévios que abordaram o mesmo tema.^(^ ^1^ ^)^

Os critérios de inclusão são os seguintes^(^ ^2^ ^)^: indivíduos assintomáticos respiratórios; ter idade entre 55 e 74 anos; ser fumante com história tabágica de pelo menos 30 anos-maço ou ser ex-tabagista há no máximo 15 anos; e aceitar participar da pesquisa assinando o termo de consentimento livre e esclarecido. Os critérios de exclusão incluem os indivíduos que não consigam realizar o exame tomográfico, gestantes, aqueles submetidos à radioterapia prévia na região torácica ou que tenham doenças crônicas severas, como doenças cardiovasculares, pulmonares, hepáticas, renais ou metabólicas.

O desfecho primário analisado é o diagnóstico precoce de CP. Contudo, também é realizada uma avaliação multidisciplinar para o diagnóstico diferencial de outras patologias relacionadas ao vício tabágico e doenças infecciosas comuns em nosso meio, como a tuberculose.

Na visita inicial, são coletados dados demográficos e história tabágica, além da avaliação da qualidade de vida relacionada à saúde por meio do *Medical Outcomes Study 36-item Short-form Health Survey* (SF-36),^(^ ^3^ ^)^ bem como da avaliação da possível presença de ansiedade ou depressão por meio da *hospital anxiety and depression scale**^(^* ^4^ ^)^ e da dependência nicotínica em indivíduos fumantes atuais por meio do teste de Fagerström.^(^ ^5^ ^)^

Após completar a avaliação inicial, o indivíduo é encaminhado para a investigação radiológica por TCBD, analisada por dois médicos radiologistas com experiência em doenças do tórax. A presença de um nódulo pulmonar indeterminado = 4 mm é avaliada por uma junta médica, composta por radiologistas, pneumologistas e cirurgiões torácicos, para a tomada de decisão de acompanhamento (Quadro 1).

**Quadro 1:** Descrição das opções de acompanhamento dos pacientes de alto risco em relação à presença de nódulos sólidos, opacidade em vidro fosco e nódulos não sólidos, com base nas recomendações do National Comprehensive Cancer Network Guidelines for Lung Cancer Screening e da Fleischner Society.a TCBD: TC de baixa dosagem; e PET-TC: positron emission tomography-TC (TC por emissão de pósitrons combinada com TC). aAdaptado de National Comprehensive Cancer Network,(7) MacMahon et al.(8) e Patel et al.(9)

Para os nódulos sólidos > 8 mm, as características radiográficas avaliadas isoladamente não são capazes de distinguir entre os nódulos benignos e malignos. Portanto, torna-se importante estimar a sua probabilidade clínica de malignidade, estimativa essa conhecida como cálculo "pré-teste", que ajuda a reduzir a variabilidade interobservador quanto à probabilidade de malignidade. Nesse estudo em desenvolvimento, utiliza-se o modelo de regressão logística multivariada desenvolvido na Clínica Mayo,^(^ ^6^ ^)^ a partir de seis variáveis preditoras independentes de malignidade (idade do paciente em anos, ser fumante ou ex-fumante, história de câncer extratorácico diagnosticado há mais de 5 anos, diâmetro do nódulo em mm, presença de espículas e localização em lobo superior).

Todos os pacientes passam por uma segunda avaliação após a realização da primeira TCBD, que inclui o registro dos achados do primeiro exame e a indicação do tipo de seguimento. Nessa consulta, os participantes tabagistas atuais são encaminhados ao programa de cessação de tabagismo. A participação no programa é estimulada, embora a adesão seja voluntária.

Os achados considerados anormais na TC são registrados em uma ficha específica, analisados pela junta de especialistas e estratificados de acordo com o nível de suspeita de neoplasia, determinando-se possibilidades de acompanhamento, conforme o detalhamento na Figura 1.

**Figura 1:** Fluxograma das possibilidades de acompanhamento. TCBD: TC de baixa dosagem; e PET-TC: positron emission tomography-TC (tomografia por emissão de pósitrons combinada com TC).

Nos eventos positivos, a TCBD inicial será utilizada para a comparação do nódulo; todos os exames são realizados com os mesmos parâmetros do momento inicial, permitindo a avaliação de possíveis mudanças.

As consultas subsequentes, referentes ao segundo ano de acompanhamento, são realizadas de acordo com o mesmo fluxograma (Figura 1), considerando-se os achados específicos para cada indivíduo no ano anterior (Quadro 1).

Os resultados das TCBD são entregues ao participante pelo médico responsável do ambulatório. A equipe médica também comunicará ao participante a suspeita ou o diagnóstico de CP.

Após a confirmação diagnóstica e a resolução cirúrgica (quando indicada), os indivíduos são encaminhados para seguimento oncológico na rede do Sistema Único de Saúde ou rede suplementar, onde também receberão terapia adjuvante conforme indicação médica.

Até o presente momento, não existem estudos sobre o rastreamento de CP por meio de TCBD em países considerados em desenvolvimento, nos quais também ocorre uma maior incidência de doenças infectocontagiosas do tórax. Esse fato levanta diversos questionamentos sobre a sensibilidade e a especificidade do método para esse fim.

Esse estudo de rastreamento por TCBD é de fundamental importância em nosso meio, pois fornecerá informações específicas para a validação do método e para seu uso local como ferramenta de rastreamento populacional do CP.
